# Diffuse myocardial fibrosis - a therapeutic target? Proof of regression at 1-year following aortic valve replacement: the RELIEF-AS study

**DOI:** 10.1186/1532-429X-18-S1-O37

**Published:** 2016-01-27

**Authors:** Thomas A Treibel, Marianna Fontana, Rebecca Kozor, Patricia Reant, Maria A Espinosa, Silvia Castelletti, Heerajnarain Bulluck, Anish N Bhuva, Steven K White, Anna S Herrey, Charlotte Manisty, James C Moon

**Affiliations:** 1 Barts Heart Centre, London, UK; 2 grid.83440.3b0000000121901201Institute for Cardiovascular Sciences, University College London, London, UK; 3 grid.412041.2000000012106639XUniversity of Bordeaux, Bordeaux, France; 4 grid.410526.40000000102777938Gregorio Marañon Hospital, Madrid, Spain

## Background

In aortic stenosis (AS), LVH occurs due to cellular hypertrophy and extracellular matrix expansion (diffuse fibrosis). After aortic valve replacement (AVR) early regression has been shown by extracellular volume fraction (ECV) measurement to be cellular regression at 6 months, but diffuse fibrosis regression, predicted by one year, has not been demonstrated non-invasively. Myocardial fibrosis is a key potential drug target for new therapies in heart failure, and non-invasive proof of fibrosis regression would be a major proof-of-concept milestone in validating this target, with CMR a key candidate technique to quantify change. We used CMR ECV measurement to track the change in cell and fibrosis volume following AVR (RELIEF-AS Study: NCT 02174471).

## Methods

123 patients with symptomatic, severe AS (AVAi 0.4 ± 0.1 cm^2^/m^2^) underwent CMR at 1.5T prior to AVR. 95 patients attended repeat CMR 1-year post-op (age 69 ± 11 years; 56% male); 5 declined, 10 patients died and 13 had pacemakers implanted. T1 mapping (ShMOLLI) was performed prior to and at 15 minutes post-contrast (Dotarem). Global ECV was derived from 3 short axis T1 maps excluding segments with infarct-pattern LGE. Fibrosis volume (LV mass * ECV) and cell volume (LV mass * [1-ECV]) were calculated.

## Results

**Figure 1:**
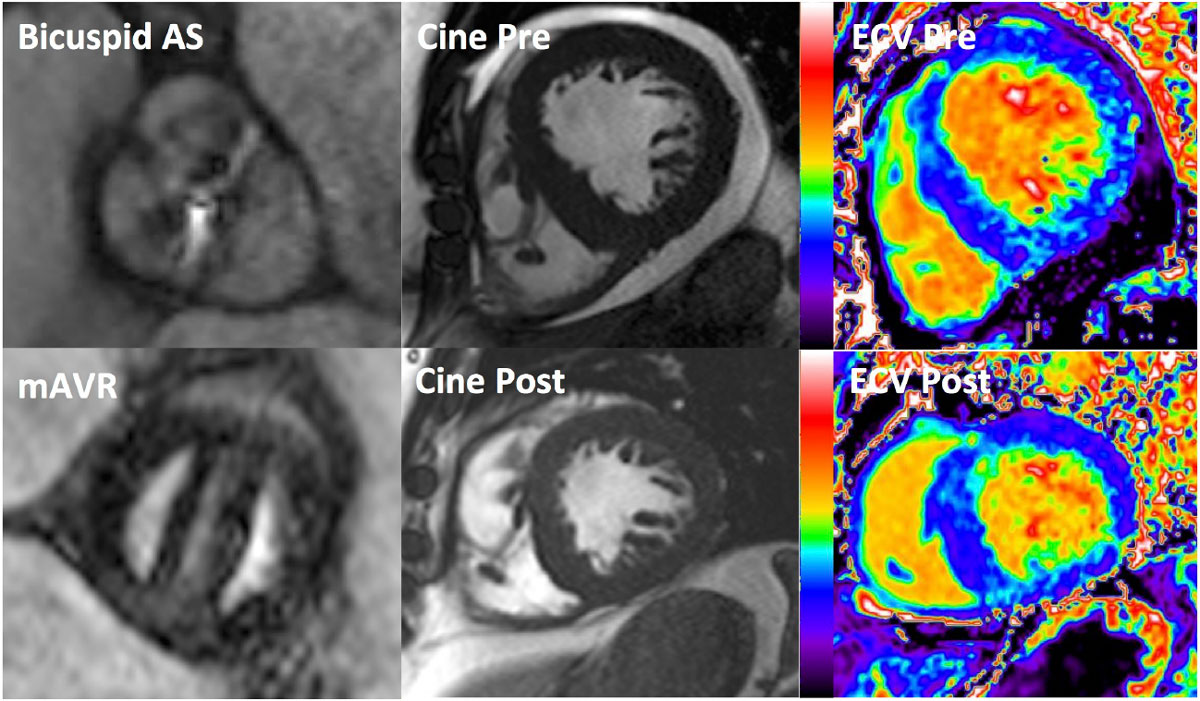
**Patient with severe AS and 1-year after mechanical AVR**. Pre-op there is severe LV hypertrophy (433 g) with LV decompensation (LVEF 32%) and pericardial effusion. At 1-year, LV mass regressed by 37% (to 273 g) with significant improvement in function (LVEF 83%). The concomitant reduction in EDV (-40%) gives the appearance of an unchanged LV geometry. ECV was unchanged at 31% resulting in a 37% reduction in fibrosis (129 to 81 mls) and cell volume (284 to 179 ml).

**Figure 2:**
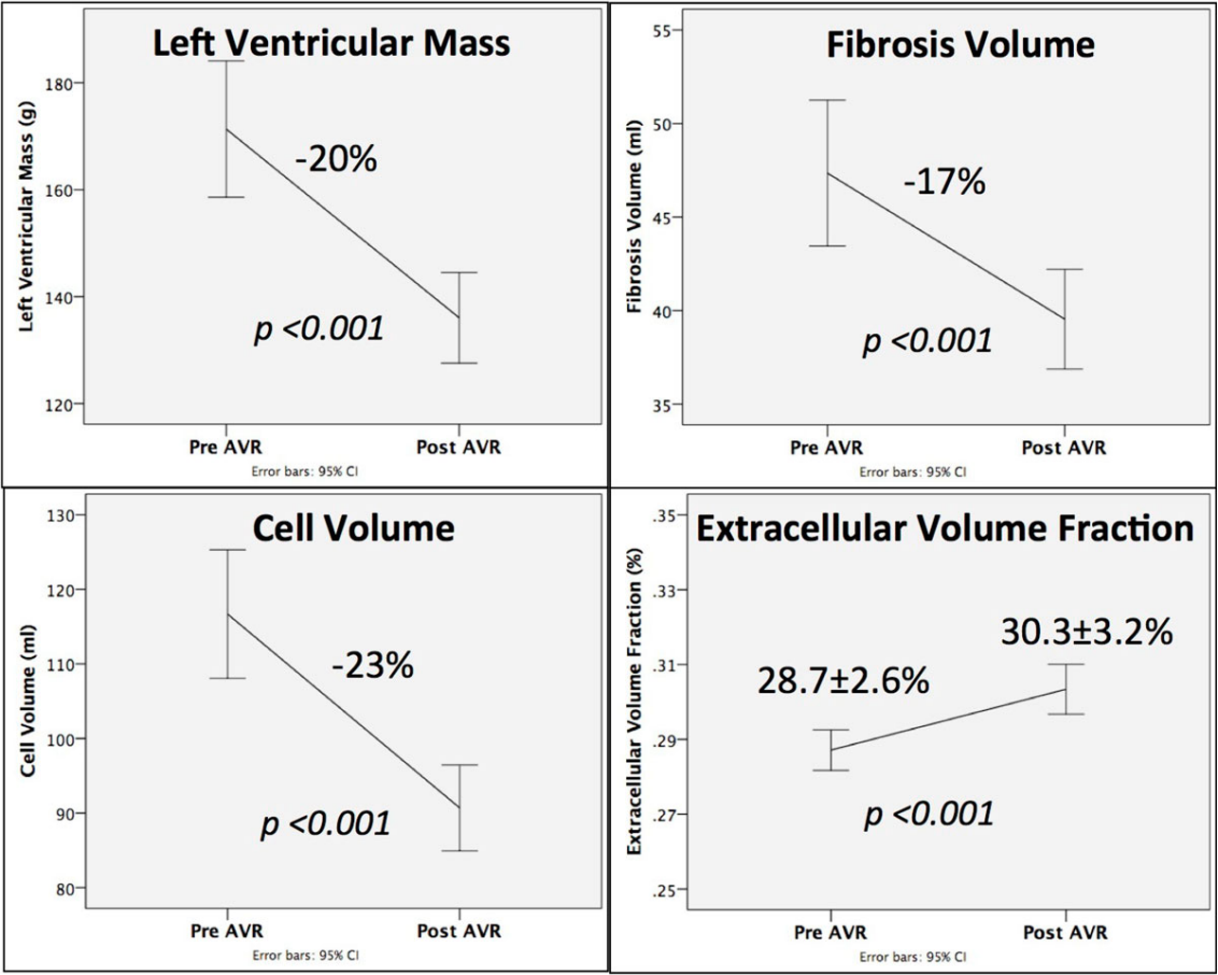
**At 1-Year Post AVR, There Is Cellular And Fibrosis Volume Regression**.

After AVR, LV mass regressed by 20% (171 ± 63 g to 136 ± 42 g, *p < 0.001*, Figure 1). Unexpectedly, ECV increased (28.7 ± 2.6% to 30.3 ± 3.2%, *p < 0.001*), which was the result of a 17% reduction in fibrosis volume (48 ± 19 ml to 40 ± 13 ml, *p < 0.001*) and a (higher) 23% reduction in cell volume (117 ± 42 ml to 90 ± 28 ml, *p < 0.001*, Figure 2). Native myocardial T1 was unchanged (975 ± 34 ms vs 971 ± 31 ms, *p = 0.6*). Mean baseline NT-pro-BNP levels declined from 174 pmol/L [IQR 29-214] to 98 pmol/L [IQR 23-126] (*p = 0.01*). Fibrosis volume reduction correlated well with NT-pro-BNP reduction (R^2^ = 0.44, *p < 0.001*).

## Conclusions

We show for the first time non-invasively that myocardial fibrosis regresses at 1-year following AVR - but less than cellular regression, so there is a small rise in ECV post AVR. These data support the position that human diffuse fibrosis is dynamic and that this is measurable by CMR - a key biological result and proof-of-concept for drug development targeting myocardial fibrosis.

